# Intensity of Nuclear Staining for Ki-67, p53 and Survivin as a New Prognostic Factor in Non*-*muscle Invasive Bladder Cancer

**DOI:** 10.1007/s12253-019-00678-1

**Published:** 2019-06-19

**Authors:** Rafał Stec, Szczepan Cierniak, Arkadiusz Lubas, Urszula Brzóskowska, Tomasz Syryło, Henryk Zieliński, Aleksandra Semeniuk-Wojtaś

**Affiliations:** 1 Oncology Department, Medical University of Warsaw, 19/25 Stępińska St., 00-739 Warsaw, Poland; 2 Patomorphology Department, Military Institute of Medicine, 128 Szaserów St., 04-141 Warsaw, Poland; 3 Internal Diseases, Nephrology and Dialysis Department, Military Institute of Medicine, 128 Szaserów St., 04-141 Warsaw, Poland; 4 General, Functional and Oncological Urology Department, Military Institute of Medicine, 128 Szaserów St., 04-141 Warsaw, Poland; 5 Oncology Department, Military Institute of Medicine, 128 Szaserów St., 04-141 Warsaw, Poland

**Keywords:** Bladder cancer, NMIBC, Ki-67, p53, Survivin

## Abstract

The aim of the study was to determine the prognostic value of expression levels of biomarkers selected on the basis of the literature: p53, Ki-67, survivin, β-catenin, E-cadherin and N-cadherin in patients with non-muscle invasive bladder cancer*.* Immunohistochemistry was performed on sections of primary papillary carcinoma of the bladder removed during transurethral resection of the tumor in 134 patients. The expression of β-catenin and E-cadherin was found in all analyzed cases and N-cadherin expression was demonstrated in 3.73% of the tissues examined. The expression of the p53 protein was confirmed in 96.27% of tissues examined. The expression of the Ki-67 protein was demonstrated in all analyzed cases. Survivin expression was found in 95.52% of the study group. Multivariate analysis confirmed the relationship between the recurrence-free survival (RFS) and the intensity of the nuclear reaction for p53 (HR 1417, 95% CI 1.001–2.007, *p* = 0.049) and survivin (HR 1.451; 95% CI 1.078–1.955; *p* = 0.014), the expression level of the Ki-67 protein expressed by the TS index (HR 1.146, 95% CI 1.116–1.823, *p* = 0.005) and the use of adjuvant BCG therapy (HR 0.218, 95% CI 0.097–0.489, *p* = 0.0002). The evaluation of Ki-67 expression and the intensity of nuclear staining for survivin and p53 may provide additional information that will allow more accurate stratification of the risk of NMIBC recurrence after TURBT.

## Introduction

The basic treatment for bladder cancer diagnosed at the stage of non-muscle invasive bladder cancer (NMIBC) is transurethral resection of the bladder tumor (TURBT). However, observational studies indicate that 48–61% of patients undergoing this treatment have a recurrence of the disease, and thereby require close monitoring for many years [1–3]. The well-known risk factors for cancer recurrence include the number and size of neoplastic lesions, the presence of coexisting carcinoma in-situ (CIS), the number of recurrences, T stage according to the TNM classification, the histopathological grade of the tumor, sex, and age [2, 4]. Based on selected risk factors, prognostic tables have been developed to determine the risk of NMIBC recurrence; however, as demonstrated by later analyses, they overestimate the risk of recurrence in some groups of patients [4, 5]. Adding new elements to existing tables may improve accuracy and, at the same time, increase the usability of prognostic models. Factors of potential prognostic significance are: p53, Ki-67, survivin, β-catenin, E-cadherin and N-cadherin.

The p53 protein is an important element regulating the cell cycle. Activation of p53 leads to the induction or inhibition of the expression of genes involved in the regulation of the cell cycle at G1 and G2 checkpoints, regulation of apoptosis, DNA repair and cell aging [6, 7]. Mutation of the *TP53* gene may cause urothelial hyperplasia and dysplasia, which are early stages of neoplastic transformation [8]. Survivin, a member of the inhibitor of apoptosis family, plays a role in the chromosomal segregation by regulating the transition from the G2 to M phase of the cell cycle. The survivin/CDK4 complex also activates the p21 protein that inhibits the apoptosis [9, 10]. Studies carried out by Zhang et al. showed that survivin silencing results in inhibition of tumor cell growth [11]. The Ki-67 protein, regarded as a proliferation marker, can be detected in the cell nuclei in the G1, S and G2 phases of the cell cycle and in mitosis. The function of this protein in the regulation of the cell cycle has not been clearly determined, but it has been shown that its presence is essential for the proliferation process [12]. E-cadherin, N-cadherin and β-catenin are involved in maintaining intercellular connections. Decreased expression of E-cadherin and increased expression of N-cadherin are elements of the epithelial-mesenchymal transition (EMT) process that increase epithelial cell migration [13]. β-catenin, which binds to actin cytoskeleton elements (a reaction mediated by cadherins), is also involved in the control of adherence and migration of cells [14, 15]. Studies on transgenic mice have shown that increased activation of β-catenin causes hyperplasia of the bladder epithelium, which may precede the development of cancer [16].

The aim of the study was to determine the prognostic value of the expression level of biomarkers selected on the basis of the literature: p53, Ki-67, survivin, β-catenin, E-cadherin and N-cadherin. The data collected in this study allow for more precise stratification of the risk of recurrence and identification of a group of patients at high risk of cancer recurrence.

## Material and Methods

### Material

The material used for the study were clinical data from patients’ medical history and sections of low- and high-grade papillary urothelial carcinoma from the Department of General, Functional and Oncological Urology of the Military Institute of Medicine in Warsaw removed during transurethral resection of the bladder tumor between 2010 and 2015. The study was approved by the Bioethics Committee of the Military Institute of Medicine in Warsaw.

All patients enrolled in the study were subjected to control cystoscopy. Patients whose follow-up period was shorter than 12 months and those with tumor recurrence within the upper urinary tract were excluded from the study. Second TURB had been performed in selected patients two-four weeks after initial resection. In those in whom neoplastic lesions were detected during the cystoscopy, TURBT was performed and the removed tissues were transferred for histopathological examination. Histopathologically confirmed papillary bladder cancer detected during the second TURB was interpreted as an incomplete resection and was not assessed as a recurrence. Each subsequent case of histopathologically proved urothelial carcinoma detected during control cystoscopy was considered as a recurrence. Second examination two-four weeks after initial resection was performed in 19 patients (14%) and 32% of them had detected bladder cancer during the second TURB.

### Immunohistochemistry

Tumor histological differentiation was graded according to the 1973 WHO classification and the assessment of the clinical stage of cancer was based on the criteria of the seventh edition of Tumor*-*Node*-*Metastasis (TNM) classification by the International Union Against Cancer (UICC, Union Internationale Contre le Cancer).

Immunohistochemistry was performed on 3-μm thick tissue sections. Monoclonal mouse antibodies (DAKO) were used to evaluate the expression of the proteins of interest (p53 – clone D0–7; Ki-67 – clone MIB1; survivin – clone 12C4; β-catenin – clone β-catenin 1 E-cadherin – clone NCH-38, N-cadherin – clone 8G11). For microscopic assessment of immunohistochemical staining for survivin, p53 and Ki-67 expression, the Allred score was used, which is the standard tool for the assessment of steroid receptor expression. The percentage of stained cell nuclei (proportion score, PS) and staining intensity (intensity score, IS) were assessed. The final result was obtained by adding the percentage of the stained cell nuclei scored on a 6-point PS scale and the intensity of staining scored on a 4-degree IS scale (Table 1) [17]. Positive protein expression was defined as any color reaction obtained (≥ 2 points in the TS scale). For microscopic evaluation of immunohistochemical staining for β-catenin, N-cadherin and E-cadherin, the scoring system was applied that is routinely used in the evaluation of HER2 receptors in breast cancer. The results obtained were classified as follows: the highest score of (3+) was defined as strong, total staining of cell membrane in more than 30% of cells. The reaction was scored as (2+) when there was a weak or moderate total staining of more than 10% of cells or a strong complete membrane staining in ≤30% of cells. The score of (1+) indicated a complete staining of not more than 10% of tumor cells or partial, incomplete staining. The result (0) indicated complete lack of expression. Positive staining for β-catenin, N-cadherin and E-cadherin was defined as obtaining color reaction of any intensity.

**Table 1:** Allred score [17]

PS	Percentage of stained cell nuclei
0	0%
1	> 0–1%
2	> 1–10%
3	> 10–33%
4	> 33–66%
5	> 66–100%
IS	Intensity of nuclear staining
0	negative
1	low
2	moderate
3	high

*PS* proportion score, *IS* intensity score

### Statistical Analysis

Statistical analysis of the results was performed using the Statistica software (StatSoft Inc.), version 12. The result of the statistical test was considered significant if the test probability p was lower than the value of type I error = 0.05. The correlations between the evaluated variables were assessed using the Spearman correlation test (rs), adequately to their non-normal distribution. In order to evaluate the potential effect of the variables on recurrence-free survival, first univariate and then multivariate Cox regression proportional regression analysis was performed.

## Results

The study group consisted of 134 patients (113 men and 21 women) with papillary non-muscle invasive bladder cancer. The overall median follow-up period was 36 months (minimum 12 months; maximum 93 months). During the analyzed period, the recurrence of neoplastic disease occurred in 74 patients, which constituted 55.22% of the examined group. The median time to recurrence was 9 (range: 3–48) months. Detailed characteristics of the study participants are presented in Table 2.

**Table 2:** Clinical and pathological characteristics of the study population

Feature	Number of patients (*n*)	Percentage (%)
Staging		
Tis	9	6.72%
Ta	51	38.06%
T1	25	18.66%
Not known	49	36.57%
Coexisting Tis		
Yes	4	2.99%
No	80	59.7%
Not known	50	37.31%
Grading		
G1	55	41.04%
G2	64	47.%
G3	14	10.45%
Not known	1	0.75%
Number of neoplastic lesions		
1	86	64.18%
2–3	29	21.64%
= > 4	19	14.18%
Diameter of the largest lesion		
< 1 cm	5	3.73%
1–2.5 cm	66	49.25%
≥ 3 cm	54	40.3%
Not known	9	6.72%
BCG therapy after cancer diagnosis		
Yes	31	23.13%
No	103	78.87%

*Tis* intraepithelial carcinoma, *Ta non-invasive papillary* carcinoma, *T1 tumor* invades subepithelial connective tissue, *G1* well differentiated cancer, *G2* moderately differentiated cancer, *G3* poorly differentiated cancer, *BCG* Bacillus Calmette-Guérin

### Immunohistochemical Assessment of Protein Expression Levels

**Fig. 1:**
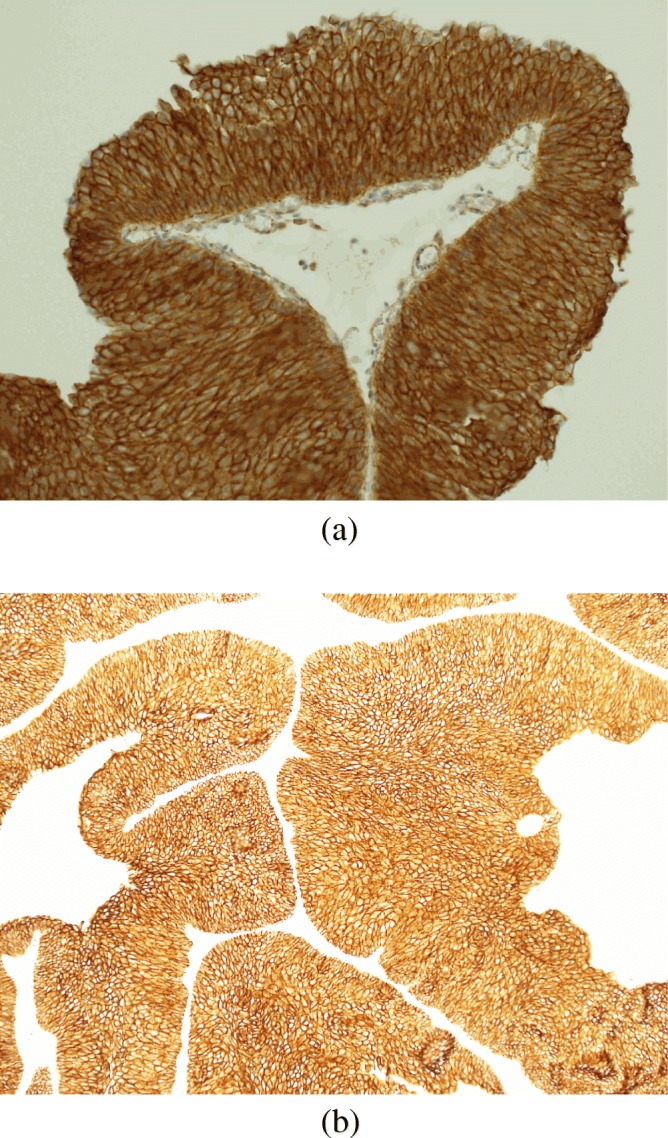
**a**. Bladder cancer – an example of positive staining anti-β-catenin antibodies (×200); membrane staining score 3+. **b**. Bladder cancer – an example of positive staining with anti-E-cadherin antibodies (×200); membrane staining score 3 +

**Fig. 2:**
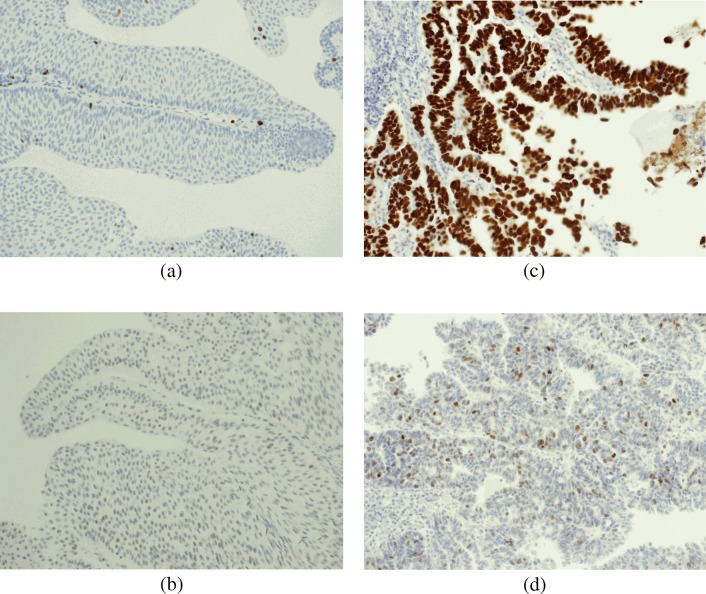
**a**. Bladder cancer – an example of positive staining with anti-Ki-67 antibodies (×200); IS2, PS1. **b**. Bladder cancer – an example of positive staining with anti-p53 antibodies (×200); IS1, PS3. **c**. Bladder cancer – an example of positive staining with anti-p53 antibodies (×200); IS3, PS5. **d**. Bladder cancer – an example of positive staining with anti-survivin antibodies (×200); IS3, PS3

The results of immunohistochemical tests for β-catenin, N-cadherin and E-cadherin are presented in Table 3. Representative photographs of positive color reaction are shown in Fig. 1. The results of immunohistochemical tests for p53, Ki-67 and survivin proteins are presented in Table 4. Representative photographs of positive color reaction are shown in Fig. 2. The expression of the p53 protein was confirmed in 96.27% of the tissues examined; the percentage of stained cell nuclei in all tissues examined was over 1%. The intensity of nuclear staining was low in 13.43% of cases, intermediate in 58.21% cases and high in 24.63% of cases. The expression of the Ki-67 protein was demonstrated in all analyzed cases. In 6.72% of patients, the percentage of stained cell nuclei was less than 1%. The intensity of stained cell nuclei was low in 5.22% of cases, intermediate in 5.97% of cases and high in the remaining 88.81% of cases. Survivin expression was found in 95.52% of the study group. In 26.12% of patients, the percentage of stained cell nuclei was less than 1%. The intensity of the nuclear staining was low in 39.55% of patients, intermediate in 42.54% of patients and high in the remaining 13.43% of patients.

**Table 3:** Immunohistochemical staining for β-catenin, N-cadherin and E-cadherin

Protein	Expression level	Number of patients (*n* = 134)	Percentage (%)
β-catenin	1	0	–
	2	6	4.48%
	3	128	95.52%
N-cadherin	No expression	129	96.27%
	1	2	1.5%
	2	3	2.23%
	3	0	–
E-cadherin	1	0	–
	2	0	–
	3	134	100%

**Table 4:** Distribution of total score for p53, Ki-67 and survivin expression levels

Expression level (TS)	P53		Ki-67		Survivin	
	Number of patients (*n* = 134)	Percentage	Number of patients (*n* = 134)	Percentage	Number of patients (*n* = 134)	Percentage
0	5	3.7%	0	–	6	4.47%
2	0	–	5	3.7%	35	26.12%
3	6	4.48%	3	2.34%	23	17.16%
4	19	14.2%	11	8.21%	40	29.85%
5	57	42.54%	40	29.85%	19	14.18%
6	26	19.4%	51	38.06%	3	2.24%
7	12	8.96%	23	17.16%	4	2.99%
8	9	6.72%	0	–	4	2.99%

*TS* total score

### Correlations Between p53, Ki-67 and Survivin Expression Levels and Clinical and Pathological Variables

Correlations of p53, Ki-67 and survivin expression levels with histopathological malignancy, the depth of bladder wall invasion by the tumor, and the diameter and number of tumor lesions were assessed. The evaluation of the correlations for the expression of β-catenin, N-cadherin and E-cadherin could not be performed due to the very low variability of traits within the studied population.

The intensity of nuclear staining for p53 correlated with the number of neoplastic lesions found during cystoscopy (rs = 0.189) and with the histopathological differentiation of the tumor (rs = 0.176). The PS index for the p53 protein correlated with the diameter of the largest neoplastic lesion (rs = −0.227). When analyzing the expression of the Ki-67 protein, a statistically significant relationship was found between the PS (rs = 0.352) and TS indices (rs = 0.292) and the histopathological grade of the tumor. Survivin expression showed statistically significant correlation with tumor histopathology (TS: rs = 0.387, IS: rs = 0.258, PS: rs = 0.333) and the depth of bladder wall invasion (TS: rs = 0.280; IS: rs = 0.228; PS: rs = 0.260).

### Assessment of the Prognostic Value of 53, Ki-67 and Survivin Expression Levels in Non-muscle Invasive Bladder Cancer

Survival analysis using Cox proportional regression model showed a statistically significant relationship between recurrence-free survival (RFS) and the intensity of the nuclear reaction for p53 (*p* = 0.001), survivin (p = 0, 12) and Ki-67 proteins (*p* = 0.048); the percentage of stained cell nuclei for Ki-67 (*p* = 0.0004); expression levels of survivin and Ki-67 expressed as TS index (*p* = 0.029 and *p* = 0.0001); the diameter of the largest neoplastic lesion (*p* = 0.002); and the use of BCG adjuvant therapy (*p* = 0.0007). Multivariate analysis confirmed the relationship between RFS and the intensity of nuclear reaction for the p53 protein (HR 1.417, 95% CI 1.001–2.007, *p* = 0.049) and survivin (HR 1.451; 95% CI 1.078–1.955; *p* = 0.014); Ki-67 protein expression level expressed by TS index (HR 1.146, 95% CI 1.116–1.823, *p* = 0.005); and the use of BCG adjuvant therapy (HR 0.218, 95% CI 0.097–0.489, *p* = 0.0002). The correlations between the above variables are shown in Table 5. The features determined by the EORTC (European Organization for Cancer Research and Treatment) did not show a statistically significant relationship with RFS in our population (Table 6). We also inserted a new prognostic factors to EORTC model and it seems that the evaluation of the intensity staining of nuclear reaction for p53, Ki-67 and survivn may improve the predictive value of the system (Table 7).

**Table 5:** Relation between recurrence-free survival and selected clinical and pathological variables

	Univariate analysis			Multivariate analysis		
	HR	95% confidence interval	p	HR	95% confidence interval	p
Number of lesions 1; 2–3; 4–7, >8	2.100	1.317–3.349	0.002	1.299	0.998–1.692	0.052
p53 IS	1.774	1.247–2.528	0.001	1.417	1.001–2.007	0.049
Survivin IS	1.439	1.082–1.914	0.012	1.451	1.078–1.955	0.014
Survivin TS	1.145	1.013–1.293	0.029	–	–	–
Ki-67 IS	1.984	1.007–3.9.09	0.048	–	–	–
Ki-67 PS	1.602	1.234–2.08	0.0004	–	–	–
Ki-67 TS	1.603	1.260–2.038	0.0001	1.146	1.116–1.823	0.005
Adjuvant BCG therapy	0.28	0.134–0.583	0.0007	0.218	0.097–0.489	0.0002

*IS* intensity score, *PS* proportion score, *TS* total score, *BCG* Bacillus Calmette-Guérin, *HR* hazard ratio, *p* statistical significance level, − not assessed

**Table 6:** Influence of EORTS conditions on NMIBC recurrence

EORTS conditions	Hazard ratio (HR)	− 95% CI (HR)	+ 95% CI (HR)	*p* value
Number of tumors	1.948	1.168	3.247	0.011
The largest tumor diameter	3.180	1.663	6.080	< 0.001
Tumor grade	1.167	0.710	1.916	0.543
T-category	0.777	0.367	1.646	0.511
Concomitant CIS	0.665	0.199	2.226	0.508

**Table 7:** Effects of the addition of the new prognostic factors to EORTS system

System	R^2^	SBC	AIC
EORTS	0.369	321.6	312.9
EORTS + IS KI67	0.386	324.2	313.7
EORTS + IS Survivin	0.432	321,0	310.5
EORTS + IS p53	0.479	317.3	306.9
EORTS + IS KI67 + IS Survivin + IS p53	0.531	320.3	306.4

*AIC* Akaike’s Information Criteria, *SBC* Schwartz’s Bayesian Criterion

## Discussion

The commonly accepted risk factors of NMIBC recurrence do not allow for precise estimation of the risk of recurrence; therefore, markers are sought for identification of high-risk patients. This study evaluated the expression of proteins involved in the pathogenesis of papillary carcinoma of the bladder and their significance in relation to selected clinical and pathological factors.

Positive staining for β-catenin and E-cadherin was obtained in all analyzed patients. The percentage of NMIBC patients with positive β-catenin staining in published studies was lower than that obtained in the presented study – 75% in the study by Elsherif et al., 60% in the study by Koletsas et al. and 40% in the study by Senol et al. [18–20]. The obtained results of E-cadherin assays are confirmed by reports by Singh et al., whereas studies performed by Muramaki et al. and Abufaraj et al. showed the expression of E-cadherin in 50–60% of analyzed tissues [21–23]. The expression of N-cadherin, which indicates that the cell acquires migratory abilities, was only visible in 3.73% of participants of this study. Similar results were obtained by Singh et al. who observed a positive staining for N-cadherin in 13.3% of cases [21]. These data differ from the results reported by Muramaki et al. and Abufaraj et al. who confirmed the expression of N-cadherin in 40% of cases [22, 23]. As for Ki-67 expression, the result of immunohistochemical tests obtained in our study was similar to that of the Chen et al.’ study in which low Ki-67 expression was confirmed in 53% of cases and high in the remaining 47% [24]. In our study, p53 expression was found in more than 95% of patients, and in 30% of cases the percentage of stained cell nuclei was over 33%. Similar results were obtained by Senol et al. and Vetterlein et al. who showed positive staining in 30–40% of cases [19, 25]. The level of survivin expression in our patients was consistent with the results obtained by Xi et al. who confirmed protein expression in 84.5% of cases [26]. Different data were reported by Skagias et al., Jeon et al. and Senol S. et al., who observed protein expression in 61%, 48.2% and 37% of cases [19, 27, 28], respectively.

Discrepancies between the above data may result from various characteristics of patients included in the study due to genetic differences between Ta and T1 tumors. In our study, the percentage of patients with stage Ta cancer was several times higher than that of patients with T1 tumors (40% and 20%, respectively). In the quoted studies performed by other authors, patients with T1 tumors constituted 40–60% of the examined group [18, 22, 23]. In the study by Koletsas et al., patients with muscle invasive bladder cancer were also included in the analysis [20]. There were also differences regarding the interpretation of color reactions. In our study, the maximum score in the evaluation of membrane staining for protein expression was given for total staining of the cell membrane in at least 30% of cells, and in the study by Singh et al. the expression of E-cadherin was considered high if the percentage of stained cells exceeded 67% [21]. In the studies quoted above, a positive result for p53 was staining of at least 80% of cells and a positive result for survivin – at least 10% of cells, whereas in our study any color reaction was considered positive [19, 25, 27]. Differences in the results obtained may be a consequence of the use of different sets of diagnostic antibodies because, as shown by analyzes performed by van de Vijver, the percentage of positive responses may range from 2 to 30% depending on the set of antibodies used [29].

Statistical analysis showed a significant relationship between the expression of proteins involved in the regulation of the cell cycle and the clinical and pathological features of the tumor. The expression level of the Ki-67 protein expressed by the percentage of stained cell nuclei correlated with the degree of histopathological differentiation of the tumor, which is consistent with data from the literature [30–32]. This study did not confirm the relationship between Ki-67 expression and bladder wall invasion; neither did the study performed by Abdelkri et al. [93]. This relationship was, however, demonstrated in the studies of Wang et al. and Thakur et al. which also included patients in whom the tumor invaded the muscle wall of the bladder [30, 32]. The analysis of the p53 protein expression showed a statistically significant relationship between the histopathological differentiation of the tumor and the percentage of stained cell nuclei and staining intensity, which is consistent with literature reports [19, 25, 33]. Different results were obtained by Wang et al., which may be due to differences between the analyzed groups [30]. Our own study also showed a relationship between the level of the p53 protein expression and the number and size of neoplastic lesions – the assessment of this relationship is not reported by the authors cited, but may confirm the important role of this protein in the development of bladder cancer. The evaluation of the survivin expression confirms data from the literature, which suggests the association of survivin, a protein involved in the inhibition of apoptosis, with the pathogenesis of bladder cancer [26–28]. However the results of a meta-analysis performed by Lv et al. did not confirm a statistically significant relationship between protein expression and histopathological grade of neoplasm, which indicates the need for further research in this area [34].

The present study confirmed the prognostic significance of known risk factors for tumor recurrence: the diameter of the largest neoplastic lesion (*p* = 0.001) and lesions found during cystoscopy (*p* = 0.01). A statistically significant effect on recurrence-free survival was also demonstrated for expression levels of p53, Ki-67 and survivin. The demonstrated relationship between the recurrence-free survival and the survivin expression is consistent with the results of the meta-analysis by Lv et al. (HR 1.831, 95% CI, 1.344–2.493, *p* = 0.009) [34]. Our study also confirms the analyzes performed by He et al. which revealed the relationship between the level of Ki-67 protein expression and the recurrence-free survival in the Caucasian population (HR 1.441, 95% CI, 1.014–2.047) [35]. The results of presented study differ from those of the meta-analysis by Zhou et al. who did not show a significant relationship between p53 expression and recurrence-free survival in BCG-treated patients (HR 1.400, 95% CI: 0.910–2.160) [36]. The difference between the obtained results may be related to, among other things, various methodologies used in the discussed studies (in our study, protein expression was determined by assessing the staining intensity, while in the Zhou’s meta-analysis the level of p53 expression was categorized by the percentage of stained cell nuclei).

Our investigated group of patients differs significantly from this considered in EORTS. The main reason of this discrepancy is that we included patients with primary NMIBC in opposite to patients with recurrent NMIBC considered in EORTS (system based on data containing the number of tumors, the largest tumor diameter, prior recurrence rate, T category, the presence of concomitant CIS and tumor grade). Thus one of the six conditions could not be assessed. Our observation is confirmed by a lack of significant influence on time to tumor recurrence of some EORTS conditions in our study (Table 6). Nevertheless, the inclusion of any of the proposed new prognostic factors to the EORTS system seems to improve this tool, because the higher R^2^, the higher influence on the prediction variability. On the other hand, the model is better if AIC (Akaike’s Information Criteria) and SBC (Schwartz Bayesian Criterion) are lower because these indexes penalize the inclusion of additional variables to a model (Table 7).

The study has several limitations. It was a single-center, retrospective study in which the prognostic value of selected proteins was evaluated on a small group of patients. In addition, included in the study were only patients who underwent TURBT and were qualified for adjuvant BCG therapy. Nevertheless, the applied assessment system allowed for a precise evaluation of the expression of the proteins of interest and the identification of variables of significant importance in the assessment of the recurrence probability.

## Conclusion

Evaluation of the expression of Ki-67, p53 and survivin proteins provides additional information on tumor aggressiveness and may allow more accurate stratification of the risk of cancer recurrence. However, further studies assessing the prognostic value of these proteins are needed.

### Clinical Practice Points

1. The observational studies indicate that 48–61% of non-muscle invasive bladder cancer patients undergoing transurethral resection of the tumor have a recurrence of the disease, and thereby require close monitoring for many years.
2. The commonly accepted risk factors of recurrence do not allow for precise estimation of the risk of recurrence; therefore, markers are sought for identification of high-risk patients.
3. The evaluation of Ki-67 expression and the intensity of nuclear staining for survivin and p53 may provide additional information that will allow more accurate stratification of the risk of NMIBC recurrence after TURBT.
